# Experimental and Theoretical Study of an Autowave Process in a Magnetic Fluid

**DOI:** 10.3390/ijms23031642

**Published:** 2022-01-31

**Authors:** Vladimir Chekanov, Anna Kovalenko

**Affiliations:** 1 Department of Digital Development, North-Caucasus Federal University, 1 Pushkin Street, 355017 Stavropol, Russia; 2 Department of Information Technologies, MIREA-Russian Technological University, 8 Kulakova Avenue, 355000 Stavropol, Russia; 3 Department of Data Analysis and Artificial Intelligence, Kuban State University, 149 Stavropolskaya Street, 350040 Krasnodar, Russia; savanna-05@mail.ru

**Keywords:** autowaves, magnetic fluid, liquid membrane, nanoparticles, mathematic modeling

## Abstract

Magnetic fluid (MF) is a colloidal system consisting of ferromagnetic particles (magnetite) with a diameter of ~10 nm suspended in a dispersion medium of a carrier fluid (for example, kerosene). A distinctive feature of magnetic fluid is the fact that when an electric field is applied to it using two electrodes, thin layers consisting of close-packed particles of the dispersed phase are formed in the regions near the surface of both electrodes. These layers significantly affect the macroscopic properties of the colloidal system. In this work, the interpretation of the near-electrode layer is for the first time given as a new type of liquid membrane, in which the particles of the dispersed phase become charged with the opposite sign. On the basis of experimental studies, we propose a physicochemical mechanism of the autowave process in a cell with a magnetic fluid. It is based on the idea of oppositely recharging colloidal particles of magnetite in a liquid membrane. A mathematical model of an autowave process, which is described by a system of coupled partial differential equations of Nernst–Planck–Poisson and Navier–Stokes with appropriate boundary conditions, is proposed for the first time. One-dimensional, two-dimensional, and three-dimensional versions of the model are considered. The dependence of the frequency of concentration fluctuations on the stationary voltage between the electrodes was obtained, and the time of formation of a liquid membrane was estimated. Qualitative agreement between theoretical and experimental results has been established.

## 1. Introduction

**Figure 1:**
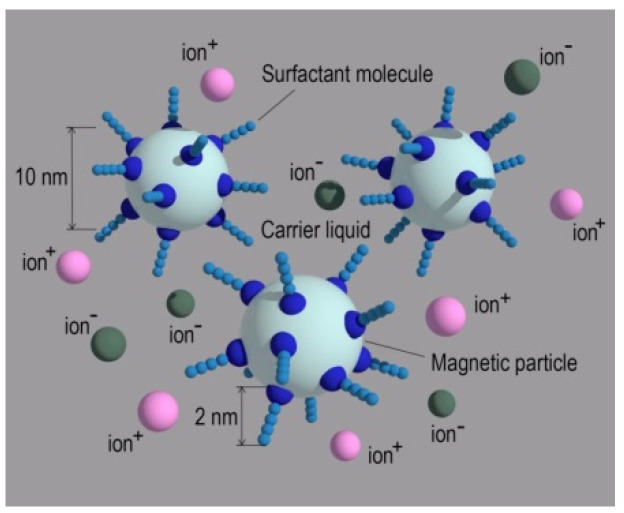
Schematic representation of a magnetite fluid: colloidal particles (Fe_3_O_4_ nanocrystals) in a carrier liquid (kerosene) stabilized by surfactant molecules (oleic acid); ions+, ions—(impurity ions). Impurity ions (with a concentration of 10^20^ m^–3^) appear during the preparation of MF, their amount depends on the quality of the dispersed magnetite washing with the deionized water.

Magnetic fluids (MF) are ultrafine stable colloids of ferro- or ferrimagnetic single-domain particles dispersed in various liquids: water, organosilicon compounds, or hydrocarbons. Colloidal particles of iron, cobalt, nickel, and ferromagnetic oxides are used as the dispersed phase [1]. To prevent the coagulation of a colloidal solution due to magnetic dipole–dipole and Van der Waals interactions with the subsequent enlargement of particles, surfactants are used as stabilizers [2]. Being adsorbed on the surface of dispersed particles, surfactants form a protective shell, which works as a structural and mechanical barrier. As a result, MF does not delaminate and retains its homogeneity for an almost unlimited time (Figure 1).

The aggregate stability of magnetic fluids, the high dispersion of the magnetic phase, a unique combination of fluidity, and the ability to interact with magnetic and electric fields allow the use of magnetic fluids in various fields. Due to their physicochemical properties, nanoparticles of the “magnetic core–shell” type demonstrate high adsorption efficiency and a high rate of removal of pollutants, as well as easy and quick separation of the adsorbent from the solution using an external magnetic field, which allows them to be used in nature-saving technologies—the collection of oily contaminants in water and the removal of organic waste from water with their subsequent catalytic processing [3]. Recently, magnetic fluids have been proposed to convert thermal gradients into electromagnetic energy by exploiting induction and thermomagnetic advection [4]. Due to low intrinsic toxicity and ease of synthesis, as well as high indicators of physical and chemical stability, MFs have found application in medicine using the methods of hyperthermia, biological analysis, and catalysis [5,6]. Such properties of magnetic colloids as high magnetocrystalline anisotropy, high coercive force, moderate saturation magnetization, and good chemical and structural stability at high temperatures have proven to be very useful in industry for infrared transmission spectrometry and thermogravimetry, the synthesis of metal oxide nanoparticles, and space technologies [7,8,9].

Magnetic fluid reveals effects that are interesting for practical application when interacting not only with a magnetic but also with an electric field. For example, MF is used in magnetic fluid control elements in hydraulic systems. Moreover, it is used as an active medium in electrical devices and apparatuses, for example, in an induction neutralizer of static electricity. This method is non-sparking, which allows it to be used in oil refining, electronics, and other industries. Non-sparking potential equalization on isolated structures is an urgent problem in space technologies [7,10,11].

The most widespread and aggregately stable MFs are based on magnetite (Fe_3_O_4_) dispersed in hydrocarbon and organosilicon liquids and water. In this work, we investigate the liquid “magnetite in kerosene” with oleic acid as a stabilizer, which is a weakly conducting dielectric with a conductivity of about 10^−7^ Ohm^−1^/m^−1^. The passage of the electric current is provided by impurity ions (with a concentration of about 10^20^ m^−3^) and low-mobile colloidal particles [12,13]. This process is accompanied by a disturbance in the equilibrium between the reactions of the dissociation–recombination of impurity ions. As a result of this disturbance, the nonequilibrium regions of the space charge appear at the electrodes [12,13,14]. Charged electrodes attract colloidal particles of opposite charge sign to themselves and form near-electrode layers—colloidal systems with a high concentration of magnetic particles. The near-electrode layer does not mix with the other liquid in the cell and can be considered as a liquid membrane [15,16,17].

**Figure 2:**
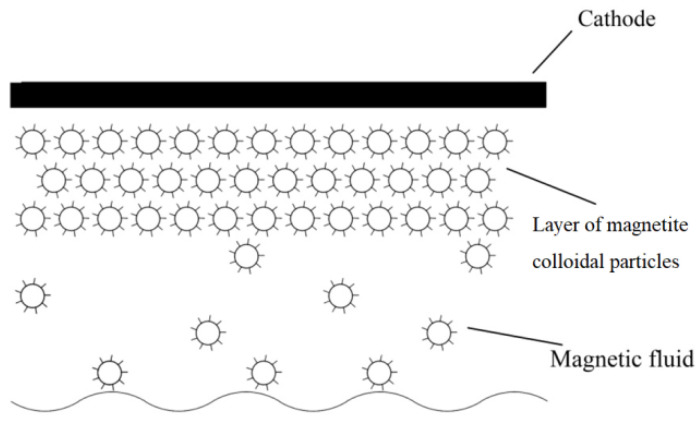
Schematic of a half of an MF cell with a layer of concentrated liquid formed in an electric field near the electrode. A similar picture applies to the anode.

The presence of magnetic particles in the near-electrode regions has a significant influence on the electrophysical and optical effects, which are specific for magnetic fluids and are not observed in other liquid dielectrics. For example, when a cell with magnetic fluid is illuminated with light with a wide spectrum of wavelengths in an electric field, we see a change in the color of the electrode surface, which is explained by a shift in the spectrum maximum due to the formation of a near-electrode layer of particles and an increase in the optical thickness of the layer [18]. Labyrinth structures of ordered microdroplet aggregates are formed in a thin layer of a magnetic colloid [19]. In an electric field, the transparency of the magnetic colloid changes [20] and layers of close-packed particles of the dispersed phase are formed at the interface with the electrodes (the concentration of particles in the layer is ~27% vol., or 0.74·10^−2^ mol·m^−3^) [21,22,23,24] (Figure 2).

The near-electrode layer has a special property—changing the charge sign of colloidal particles, which occurs with the participation of surfactant molecules used to stabilize the dispersed system [25]. In turn, this “recharging” (here and after we call “recharging” the changing of the charge sign) of particles in the near-electrode layer is the basis of the physical mechanism for the development of self-sustained nonlinear waves (autowaves) [26]. Autowave processes are a vivid example of self-organization. The importance of studying these processes is due, among other things, to the fact that they are widespread in living organisms, for example, in the heart muscle, brain, nerve fiber, etc. Any autowave processes in physical, chemical, and biological objects have some common properties. Therefore, when studying the properties of autowaves in a magnetic fluid, new knowledge appears about this type of wave, for example, about the formation and reproduction of reverberators, the behavior of autowaves near an obstacle, etc. This knowledge can be applied in the study of processes occurring in excitable biological tissues: the myocardium of the heart, the retina of the eye, etc. Reverberators are of particular interest for research, since a number of their applications are known: in the sinoatrial node of the heart in atrial fibrillation. Although there is no direct analogy between autowave processes in a magnetic fluid and similar processes in living systems, the obtained regularities will be useful both in fundamental and applied aspects. Previously, autowaves were also experimentally studied: in the well-known Belousov–Zhabotinsky reaction, only a few tens of cycles can be observed, then the reaction stops [27]. Other examples of self-organization in heterogeneous systems that can be visually observed are described in [28]. The dynamics of self-organization processes in a thin layer of a magnetic fluid were studied in [29]. Brightly colored autowaves with all modes characteristic of autowave processes were observed in an electrochemical cell with a magnetic fluid [26]. The autowave process investigated in this paper is observed in a constant electric field. A magnetic field was also applied but did not have a significant effect on the process. Due to the electrical characteristics and optical properties of MF, the autowave process is easily reproduced in laboratory conditions and the number of observed cycles is not limited. Therefore, a cell with magnetic fluid is a good model environment for studying the autowave process. The lack of a well-defined mechanism for the development of the autowave process in MF, confirmed by modeling and numerical experiments, is a gap in previous studies in this area, as well as the lack of a model based on real processes occurring in a cell with an MF.

One of the first mathematical models of an autowave process in a magnetic fluid was the model proposed in [30]. This model consisted of a single partial differential equation of parabolic type, and the medium itself was considered as bistable. In [31], the basic FitzHugh–Nagumo model [32,33], consisting of two differential equations of parabolic type, was applied to simulate the autowave process in a magnetic fluid. The near-electrode layer of a magnetic fluid was considered as an excitable medium, which can be in three states: rest, excitement, and refractoriness. The disadvantage of this model is that it describes the processes occurring in the equivalent electrical circuit of a cell with magnetic fluid, rather than real processes.

A comprehensive experimental and theoretical study of the autowave process in MF is carried out in the current paper. A necessary condition for the development of the autowave process is the formation of a dense near-electrode layer of dispersed phase particles, which was first interpreted by the authors as a magnetic-liquid membrane with special properties. Depending on the magnitude of the applied electric field, it can vary in thickness and be destroyed at a certain critical value of the field.

The model is described by a system of coupled partial differential equations of Nernst–Planck–Poisson and Navier–Stokes with appropriate boundary conditions. The model takes into account the processes of particle recharge in a near-electrode dispersed layer. Thus, the described model is a first principle model of the autowave process in magnetic fluid, which is built on a fundamental understanding of the underlying physico-chemistry.

## 2. Discussion of the Experimental Results

### 2.1. Charge Transfer in MF

According to reviews [12,34], the electrical conductivity in MF is due to several mechanisms, including the presence of impurity ions appearing in the MF during its synthesis. In addition, colloidal magnetic particles can also transfer charge [35,36].

In the synthesis of MF, Fe_3_O_4_ magnetite nanoparticles are most often obtained by the method of chemical coprecipitation or by the method of “chemical condensation” as a result of the reaction [37] and references in this work. The method of chemical coprecipitation usually involves the precipitation of Fe^3+^ and Fe^2+^ salts in a ratio of 2:1 in an aqueous medium using strong alkalis such as NaOH and KOH in an inert atmosphere and at a low temperature (so-called Massart’s method):

FeCl_2_ + 2FeCl_3_ + 8NaOH → Fe_3_O_4_ + 8NaCl + 4H_2_O.

**Figure 3:**
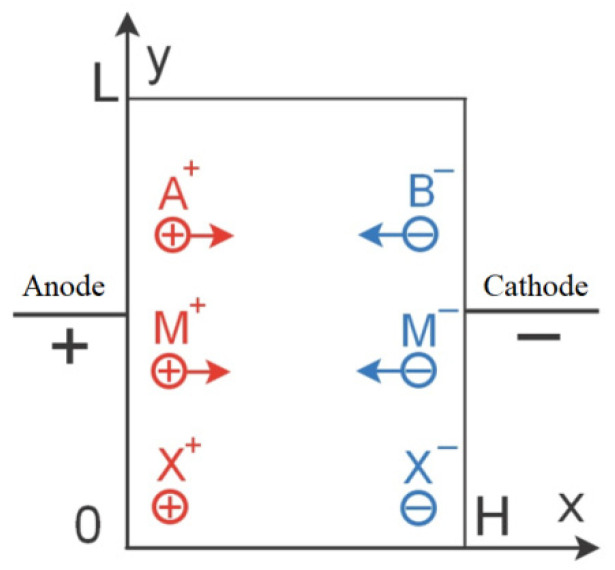
Schematic of a cell with a magnetic fluid. A^+^ and B^−^—the impurity ions; M^+^ and M^−^—the charged particles of the magnetic fluid; X^+^ and X^−^—the injected ions.

Water is removed during manufacture, but not completely. Peptization is carried out by the action of oleic acid on the sediment, then the stabilized particles are transferred into the carrier liquid. Thus, the MF can contain Na^–^, Cl^–^, etc. The amount of impurity ions depends on the quality of the dispersed magnetite washing. Usually, the concentration of all impurity ions does not exceed 10^20^ m^−3^. In our experiments, we use a liquid with a specific conductance σ = 10^−7^ Ohm^−1^·m^−1^. The contribution to the current transfer is made by impurity ions and colloidal particles of magnetite *M^+^* and *M^–^*, which, as a result of electrophoresis, move to the corresponding electrodes [36] (Figure 3).

Colloidal particles become charged in the region of the spatial near-electrode charge and as a result of electrode processes, which are also called injective [12,34,38]. In medium high-voltage fields of the order of 10^5^ V/m, electrochemical injection starts on the electrodes.

Oleic acid molecules surround a magnetite nanoparticle, thus forming a colloidal particle (micelle) [39]. The length of the oleic acid molecule is ~2 nm, so there is a layer of oleic acid molecules between the core of the colloidal particle and the electrode (Figure 1). In addition, there is always some free oleic acid in the liquid. The head of the oleic acid molecule is an electronegative group capable of attaching an electron, and in the electrochemical process a double bond is most likely broken, therefore, the electrochemical injection of charges at the cathode can be schematically represented as:

(1) $$R_{1}=R_{2}-X+{\;e}^{-}\overset{k_{\mathrm{OA}}}{\to }{\;X}^{-}+products$$

where ${\;X}^{-}$ is the electronegative group with an electron e^−^, captured from the metal surface in the course of the electrochemical reaction, and $k_{\mathrm{OA}}$ is the rate constant of the electrochemical reaction (1).

If initially positively charged colloidal particles appeared near the cathode surface as a result of the electrophoresis in an electric field, then when they come into contact with the cathode surface, these colloidal particles’ charge sign is changed: the positive charge changes to negative due to the adsorption of negative ions ${\;X}^{-}\;$ on the particles.

Similar processes, but in the opposite direction, take place at the surface of the anode, where colloidal particles of magnetite that come to this electrode, surrounded by oleic acid, change their charge to the opposite when electrons are transferred from the OA molecules to the surface of the anode. These processes take place in a dense surface layer of particles, where colloidal particles and injected ions have almost zero mobility.

### 2.2. Mechanism of the Observed Autowave Process

When the electrodes become charged and an electric field appears between them, space charge regions, formed by impurity ions, appear in the near-electrode regions [12]. The equilibrium of dissociation–recombination reactions of ion pairs, formed by liquid molecules as well as impurity ions and surfactant molecules, is disturbed in the region of inhomogeneity of the impurity ion propagation. Therefore, this region of inhomogeneity is called a nonequilibrium layer [12] (a zone of nonequilibrium), the thickness of which is designated as *ξ_d_*. Outside the nonequilibrium layer, the dissociation–recombination reaction is in equilibrium, that is, *n*_1_ = *n*_2_ = *n*_0_, while the electric field at the boundary of the nonequilibrium layer is *E*_0_. The thickness of the nonequilibrium region is determined by the formula [12]:

(2) $$\xi_{d}\approx \frac{\varepsilon \varepsilon_{0}E_{0}(b_{1}+b_{2})}{\sigma }$$

where *b*_1_ and *b*_2_ are the mobilities of the A^+^, B^–^ ions forming a nonequilibrium layer, *σ* is the conductivity of the liquid, and *E*_0_ is the electric field strength at the boundary of the equilibrium and nonequilibrium zones.

Simple estimates show that at a voltage of 10 V across the electrodes, the distance between the electrodes is 50 μm, the field strength *E*_0_ = 2.5·10^5^ V/m, the mobility of impurity ions *b*_1_ ≈ *b*_2_ ≈ 10^−8^ m^2^V^−1^s^−1^, specific conductance *σ* = 10^−7^ Ohm^−1^m^−1^, dielectric constant *ε* = 2, and the width of the space charge region *ξ*_d_ is approximately 1 μm.

In the field $\vec{E}\;$, magnetite particles acquire an induced dipole moment $\vec{p}$, in the space charge regions they become charged, and under the action of polarization and Coulomb forces begin to move towards the corresponding electrode—a layer of concentrated MF is formed, and a change in the color of the cell surface is visually observed.

The energy of attraction of the magnetic particle decreases while it goes further away from the electrode. Let us denote by δ the distance from the electrode at which the attraction energy $U_{im}^{}\;(\delta )$ is equal to the thermal energy $k_{B}T$. The membrane thickness increases until the attraction energy ($U_{im}^{}\;$) is balanced by diffusion:

(3) $$U_{im}\left(\delta \right)=k_{B}T$$

The total charge of particles in the membrane, per unit area of the membrane, induces the Coulomb repulsion energy of the membrane $U_{q}\;$, equal to:

(4) $$U_{q}=Q\delta$$

(5) $$Q\delta >\frac{1}{\delta }\int_{0}^{\delta }U_{im}\left(x\right)dx$$

When $U_{q}\;$ exceeds the average attraction energy of $U_{im}^{}\;$, the layer gets away from the electrode and the electrochemical process at the electrode stops.

**Figure 4:**
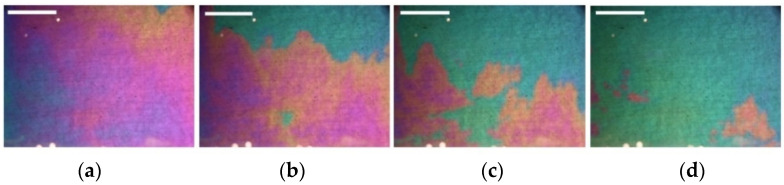
Single (“fast”) wave in the near-electrode layer (liquid membrane) of a cell with a magnetic fluid. Time passed from the process beginning: (**a**) 0 s, (**b**) 0.25 s, (**c**) 0.5 s, (**d**) 0.75 s. All scale bars are 1 cm.

Thus, under the condition $U_{q}>U_{im}\;$, a concentration wave appears in the magnetic fluid (Figure 4). After the destruction of the layer particles, pushing off from one electrode, moving to the opposite electrode, becoming recharged in the near-electrode region of the space charge, and moving to the opposite electrode again, the process is repeated.

**Figure 5:**
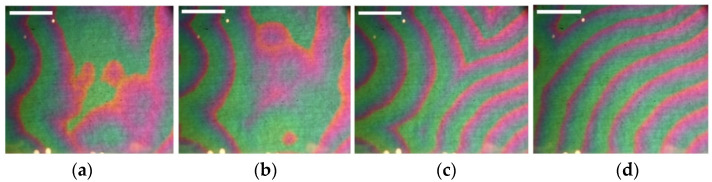
Steady-state autowave process. Time passed from the process beginning: (**a**) 4 s, (**b**) 8 s, (**c**) 12 s, (**d**) 16 s. All scale bars are 1 cm.

Estimation of the average speed of the wave front: from the appearance of green areas (Figure 4a) to the filling of the entire surface of the cell with this color takes ~0.25 s. This is the mode of “fast” autowaves, the speed of the wave is ~16 cm·s^−1^. The first wave is followed by the second, a picture of an autowave process is observed on the cell surface (Figure 5).

From the previous works mentioned in the introduction [20,21,22,23], it is known that the near-electrode layer in a cell with magnetic fluid is formed in an electric field at a certain voltage *U*_1_ between the electrodes. The thickness of this layer depends on the voltage between the electrodes and can be calculated by the method [16]. The layer does not mix with the main volume of the liquid; its properties (viscosity, dielectric constant, resistance, capacity, and others) differ from the properties of the liquid in the volume of the cell [27]. At a certain voltage *U*_2_, the near-electrode layer is destroyed. The structure of the layer is such that it “retains” the ions injected from the electrode, as a result of which the charge sign of the particles in the layer changes, and an autowave process is initiated. Due to the close arrangement of single-domain particles in the layer to each other, the possibility of long-range magnetic order arises, as shown in [40,41] and the references. The magnetic interaction of the particles contributes to the stability of the layer. All these facts allow us to consider the near-electrode layer of close-packed particles as a magnetofluid membrane with special properties.

## 3. Mathematical Model

### 3.1. Basic Equations of the Model

The model of charge transfer and the genesis of an autowave process (AWP) is described by a system of coupled partial differential equations of Nernst–Planck–Poisson and Navier–Stokes with appropriate boundary conditions. The whole set of equations is given in the Appendix A.

Note that this system of equations with the corresponding boundary conditions due to the properties of ion-exchange membranes was previously used by V.V. Nikonenko, M.Kh. Urtenov, and co-authors [42,43] in the modeling of electroconvection in electromembrane systems and showed its high adequacy, which made it possible to reveal new regularities of the electrodialysis process, to study the effect of diffusion and electroconvection on the surface of ion-selective membranes at over-limiting current modes [44]; to study the effect of a pulsed electric field on electroconvection during electrodialysis [45]; to discover a number of new phenomena, for example, the phenomenon of “breakdown in electromembrane systems” [46]; etc. The model considered in this work is distinguished by specific boundary conditions reflecting the process of the recharging of magnetite colloidal particles in the near-electrode layer of the liquid membrane.

When constructing a general mathematical model, it is necessary to take into account all the transfer mechanisms occurring in the system, which determine the movement of MF particles: diffusion, electromigration, convection. In addition, it is necessary to take into account the electrochemical processes on the electrodes, in particular, the effect of injected ions. In this regard, a simplified mathematical model, in which the transfer of positively and negatively charged magnetic particles with concentrations C_1_ and C_2_ and fluxes j_1_ and j_2_, is considered, and, without taking into account chemical reactions, was developed and investigated. Moreover, the influence of impurity and injected ions is taken into account indirectly in the boundary conditions.

For the first time, such a model does not contain adjustable parameters and is based only on the basic conservation laws. The model makes it possible to determine the main dependences of the characteristics of autowaves on the potential and other input parameters.

**Figure 6:**
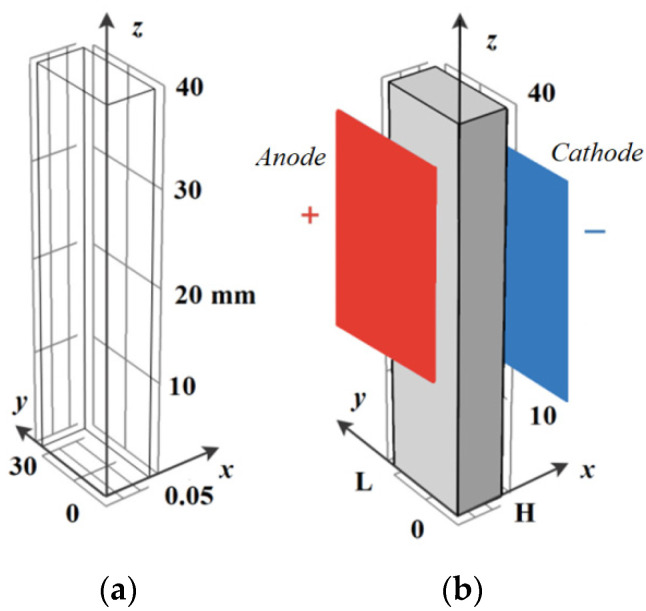
(**a**) the research area in the form of a rectangular parallelepiped, (**b**) three-dimensional diagram of a cell with a magnetic fluid (electrodes are highlighted in color).

The main idea of constructing such a model is to study the question of the possibility of the occurrence of autowave motion due to the recharging of MF particles near the electrodes or in the regions of localization of the space charge. The model is described by a boundary-value problem, which consists of a research area (Figure 6), a system of Equations (6)–(12), and initial and boundary conditions (13–20).

When constructing the model, the following assumptions were made:

1. Space charge regions are formed in the near-electrode regions of a cell with magnetic fluid in an electric field.
2. Particles are initially uncharged, their motion is due to Brownian diffusion, and they become charged only when they enter the space charge near the electrode. Moreover, only one ion is adsorbed on the particle.
3. Dense near-electrode layers consisting of particles of the dispersed phase of the magnetic fluid are formed in an electric field.
4. Impurity ions do not participate in electrode reactions because their concentration is very low. On the electrodes, the reaction occurs not due to impurity ions, but due to large organic particles (the surfactant, which is oleic acid), the concentration of which is high.

Under these assumptions, we made a model, which is described by the systems of coupled partial differential equations of Nernst–Planck–Poisson and Navier–Stokes, based on the basic physical laws of the conservation of matter and energy (6–12) and boundary conditions (13–23):

(6) $${\vec{j}}_{i}=-\frac{F}{RT}z_{i}D_{i}C_{i}\vec{E}-D_{i}\nabla C_{i}+C_{i}\vec{V},\;i=1,2,$$

(7) $$\frac{\partial C_{i}}{\partial t}=-div{\vec{j}}_{i},\;i=1,2,$$

(8) $$\varepsilon_{r}\Delta \varphi =-F\left(z_{1}C_{1}+z_{2}C_{2}\right),$$

(9) $$\vec{I}=F\left(z_{1}{\vec{j}}_{1}+z_{2}{\vec{j}}_{2}\right),$$

(10) $$\frac{\partial \vec{V}}{\partial t}+\left(\vec{V}\nabla \right)\vec{V}=-\frac{1}{\rho_{0}}\nabla P+\nu \Delta \vec{V}+\frac{1}{\rho_{0}}\vec{f},$$

(11) $$div(\vec{V})=0,$$

(12) $$\vec{f}=\rho \vec{E}$$

The Nernst–Planck Equation (6) describes the flow of charged particles of the dispersed phase of a magnetic fluid due to migration in an electric field, diffusion and convection, the charge numbers of positively charged particles $z_{1}=1$, and negatively charged particles $z_{2}=-1$. (7) Material balance equations: (8) is the Poisson equation for the potential of the electric field, (9) is the equation of current flow, $\varepsilon_{r}$ is the dielectric constant, *F* is the Faraday number, *R* is the universal gas constant, φ is the potential, $C_{i}$ is the concentration, $j_{i}$ is the flux, $D_{i}$ is the diffusion coefficient of the *i*-th charged particle, $\vec{I}$ is the current density determined by the flow of charged particles, and $\vec{V}$ is the flow rate of the magnetic fluid. Navier–Stokes Equation (10) and continuity equations for an incompressible fluid (11) describe the velocity field formed, in particular, under the action of a spatial electric force, (12) $\vec{f}$ is the density of the electric force, where $\rho =F\left(z_{1}C_{1}+z_{2}C_{2}\right)$ is the density of the space charge, $\vec{E}=-\nabla \varphi \;$ is the electric field strength, $\rho_{0}$ is the magnetic fluid density, and ν is the kinematic viscosity.

The process of the wave appearance is associated with the process of the charging and recharging of particles, which is reflected in the boundary conditions (13–23).

### 3.2. Boundary and Initial Conditions

At all boundaries of the study area, the adhesion condition is used for the velocity. The initial condition is defined as a stable solution:

(13) $$V_{x}\left(0,x,y,z\right)=V_{y}\left(0,x,y,z\right)=0.$$

The voltage on the electrodes is higher than zero.

To describe the recharge process of MF particles in a liquid membrane, we used the following boundary conditions:

At the anode (*x* = 0):

(14) $${\left(-\frac{F}{RT_{0}}D_{1}z_{1}C_{2}\frac{\partial \varphi }{\partial x}-D_{1}\frac{\partial C_{2}}{dx}\right)|}_{x=0}=j_{1A}$$

(15) $${\left(-\frac{F}{RT_{0}}D_{2}z_{2}C_{1}\frac{\partial \varphi }{\partial x}-D_{2}\frac{\partial C_{1}}{dx}\right)|}_{x=0}=j_{2A}$$

At the cathode (*x* = *H*):

(16) $${\left(-\frac{F}{RT_{0}}D_{1}z_{1}C_{2}\frac{\partial \varphi }{\partial x}-D_{1}\frac{\partial C_{2}}{dx}\right)|}_{x=H}=j_{1K}$$

(17) $${\left(-\frac{F}{RT_{0}}D_{2}z_{2}C_{1}\frac{\partial \varphi }{\partial x}-D_{2}\frac{\partial C_{1}}{dx}\right)|}_{x=H}=j_{2\;K}$$

Recharge conditions (14–17) mean that positive MF particles, when approaching the cathode, become recharged and turn into negative particles, and thus, the stream of positive particles turns into a stream of negative particles. The same happens with the flow of negative particles at the anode.

Let us consider these conditions—for example, (16), (17)—in more detail. As noted above, the recharging of magnetic fluid particles occurs in a liquid membrane located at the cathode and with a small thickness, due to ions injected from the cathode, which cannot “slip” through the liquid membrane and recombine with heteroions. This allows one to abstract from the thickness of the membrane and its location and represent it by the plane *x = H*. Moreover, let us assume that this plane is equipotential and negatively charged, i.e., is simultaneously a cathode (18).

Let a flow of positively charged MF particles with a concentration C_1_ approach the plane *x = H* at time *t*. In the *x = H* plane, the positively charged MF particles instantly change their charge (recharging occurs), become negative, and a flow of negatively charged MF particles instantly starts moving from the *x = H* plane (cathode) towards the *x =* 0 plane (anode). Let the concentration of positively charged MF particles with the concentration at time t in the plane *x = H* be *C*_1_, and negative ones *C*_2_.

Fluxes are not known in advance and are not specified, but are determined in the process of calculating the concentration of MF particles. Note that when calculating the flux of negatively charged particles *j*_2*K*_ at *x = H*, the concentration of positively charged particles C_1_ is used, which at time *t* instantly become negatively charged. Accordingly, when calculating the flux of positively charged particles *j_1K_* at *x = H* at time *t*, the concentration of negative particles C_2_ is used.

As noted above, the model uses the equipotentiality of the cathode and anode surfaces as the boundary conditions for the potential:

(18) φ(t,0,y,z)=αφ(t,H,y,z)=0.

Impermeability conditions are imposed on insulators:

(19) $$-\vec{n}\cdot {\vec{j}}_{i}=0,\;i=1,2\;$$

(20) $$-\vec{n}\cdot \nabla \varphi =0.$$

We set the initial conditions to be consistent with the boundary (21–23). We assume that at the initial moment of time, the particles of the magnetic fluid are concentrated at each of the electrodes (20), i.e., the values of the functions $C_{10}(0,x,y,z),\;C_{20}(0,x,y,z)$ are concentrated near the anode and cathode *x = 0* and *x = H*, respectively.

Below, in the calculations, as an example of the initial distribution of magnetic particles, we use the following functions:

(21) $$C_{1}(0,x,y,z)=C_{10}(0,x,y,z)=0.0074\cdot e^{-x/(0.01\cdot H)}\;mol\cdot m^{-3}$$

(22) $$C_{2}(0,x,y,z)=C_{20}(0,x,y,z)=0.0074\cdot e^{-(H-x)/(0.01\cdot H)}\;mol\cdot m^{-3}$$

(23) $$\varphi (0,x,y,z)=\alpha -\frac{\alpha x}{H}.$$

## 4. Discussion of the Numerical Experiments Results

### 4.1. One-Dimensional Model

Numerical studies were carried out with a wide variety of input parameters of the problem, here are shown the most interesting results: the distance between the electrodes is H = 5·10^−5^ m, the voltage varies in the range from 1 to 20 V, the calculation time is up to 10 s, and the concentration ranged from 2.5 to 5% vol.

**Figure 7:**
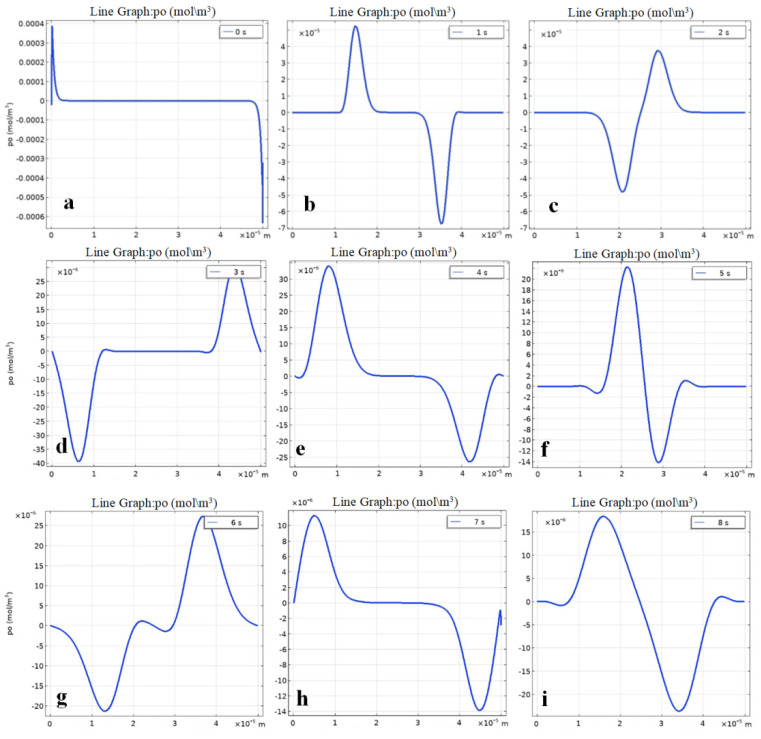
Charge density distribution in a cell with MF. (**a**–**i**) distribution of the space charge density at different moments of time. Vertical axis shows the relation of charge density to Faraday number.

**Figure 8:**
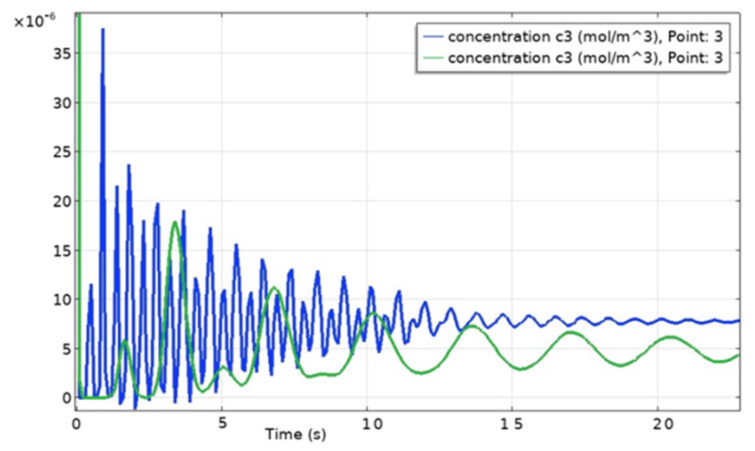
Periodic change in concentrations at *x = H* (on the cathode surface) with a voltage of 5 V (green line) and 15 V (blue line).

**Figure 9:**
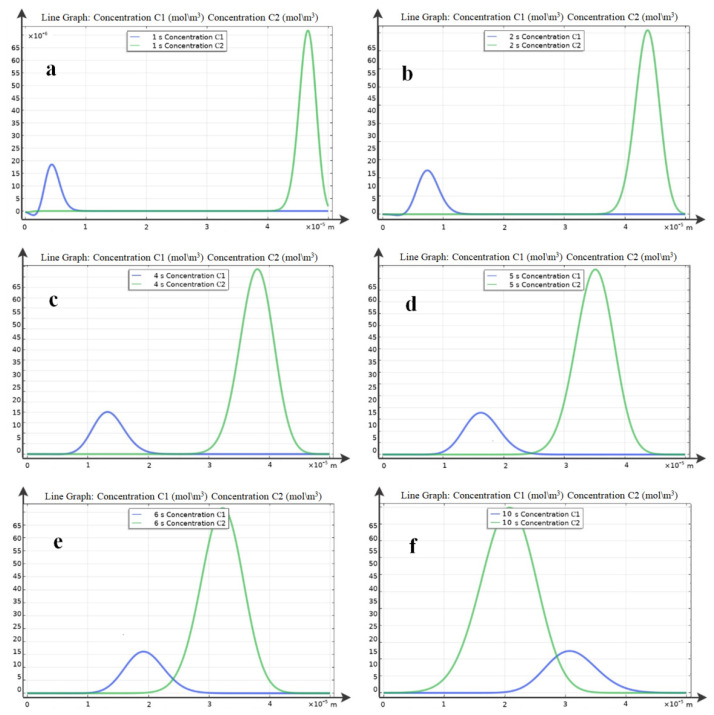
Distribution of concentrations of positively (C_1_) and negatively (C_2_) charged particles at times: (**a**) *t* = 1 s, (**b**) *t* = 2 s, (**c**) *t* = 4 s, (**d**) *t* = 6 s, (**e**) *t* = 8 s, and (**f**) *t* = 10 s.

The results of a numerical study of the one-dimensional model (24–26) with boundary conditions (13–23) are presented in Figure 7, Figure 8 and Figure 9. Figure 7 shows the distribution of the space charge density at different moments of time. Figure 8 shows the total concentration of charged particles, as well as the concentration of positively and negatively charged particles MF at moments of time *t* in Figure 9.

(24) $$\frac{\partial C_{i}}{\partial t}=-\frac{\partial j_{i}}{\partial x},\;i=1,2$$

(25) $$j_{i}=-\frac{F}{RT_{0}}z_{i}D_{i}C_{i}\frac{d\varphi }{dx}-D_{i}\frac{dC_{i}}{dx},\;i=1,2$$

(26) $$\frac{d^{2}\varphi }{dx^{2}}=-\frac{F}{\varepsilon_{r}}(z_{1}C_{1}+z_{2}C_{2})$$

The initial section (Figure 7a) characterizes the initial distribution of the space charge density after the formation of the near-electrode concentrated layer. Further, the charged MF particles begin to move towards each other (Figure 7b), this is the so-called charge waves movement under the action of an external electric field. Figure 7c shows the distribution of the space charge density at the moment when the MF particles collide with each other, i.e., when positively charged particles move towards the cathode (Figure 7d) and negative ones towards the anode and become recharged (Figure 7e). Then, they begin to move towards the opposite electrode, again passing through the region of charge of the opposite sign (Figure 7f). Figure 7d shows the space charge density distribution at the moment when positively charged MF particles approach the cathode (right) and negatively charged particles approach the anode (left). The movement of charge density waves after recharging is shown in Figure 7e, which is similar to Figure 7a. The moment of the superposition of the charge density waves is shown in Figure 7c,e,f.

Note that in Figure 7, different scales are used along the ordinate axis due to the fact that the oscillation amplitude decreases due to energy dissipation during the interaction of opposite charges.

Figure 8 shows the periodic change in the concentration of particles at different voltages on the electrodes. This figure shows that the higher the voltage across the electrodes, the shorter the period T of the concentration wave, which corresponds to the laboratory experiment.

The distribution of concentrations of positively $C_{1}(t,x)$ and negatively $C_{2}(t,x)$ charged particles at time *t* was obtained as a result of numerical experiments of the one-dimensional model (Figure 9).

At time *t* = 1 s (Figure 9a), the particles are charged. Positively charged particles MF begin to move towards x=H, and negatively charged magnetic particles towards x=0 (Figure 9b,f). The green line (C_2_) shows the negatively charged MF particles that move towards the anode, and the blue line (C_1_) shows the positively charged MF particles that move towards the cathode. Initially, two concentration waves of different amplitudes move towards each other. After the concentration waves reach the corresponding electrodes, the particles become recharged. Then, the cycle is repeated.

The one-dimensional AWP model allows one to take into account the interaction of diffusion and migration and describe the process of particle recharging; however, it does not take into account convection, which can only be taken in two- and three-dimensional models. The advantage of this model is the simplicity of the calculation and interpretation of the result. In the one-dimensional model, we obtained periodic oscillations, established the dependence of the period on the voltage, and also confirmed the hypothesis of the appearance of wave motion due to the recharging of particles at the electrodes. The model contains the most general physical equations, without artificial conditions that simulate oscillatory processes in systems, such as frequency, amplitude, period, etc.

Thus, as a result of the implementation of the one-dimensional model, the autowave process was investigated and a qualitative agreement between the numerical and laboratory experiments was obtained. However, it is obvious that for a more detailed study of the process, it is necessary to make a two-dimensional model and also take into account the motion of particles under the action of convection.

### 4.2. Two-Dimensional Model

**Figure 10:**
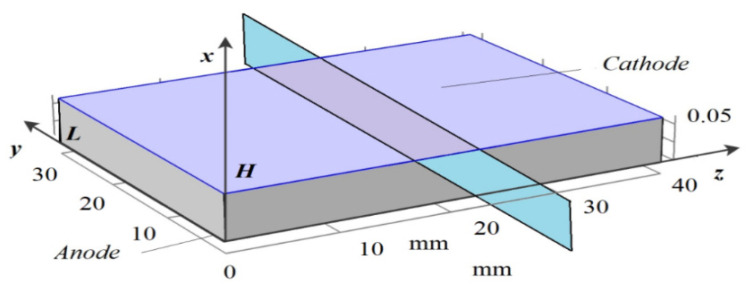
Solution domain diagram for a 2D model.

In two-dimensional modeling, we take a cross-section of a plane perpendicular to the electrodes (Figure 10), in which two opposite sides of the rectangle are electrodes and the other two sides are insulators. The coordinate system is constructed so that the anode coincides with the *yOz* plane, and the *Ox* axis is perpendicular to the anode. The cathode passes through the point *x* = *H*. (Note: *x =* 0 and *x = H* are not necessarily the locations of the anode and cathode, but conditional points in the space charge regions in which the process of charging occurs (at the point *x =* 0 there may be initially no space charge)).

**Figure 11:**
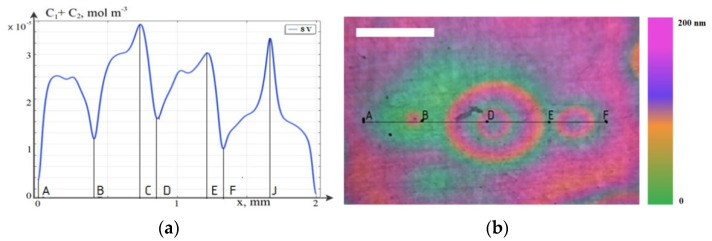
(**a**) Graph of the total concentration of C_1_ + C_2_ on the cathode surface at time *t* = 3 s. (**b**) Laboratory experiment. Concentric waves on the electrode surface, the voltage is 8 V. Color gradient shows the variable layer thickness. Scale bar is 1 cm.

The results of the direct numerical simulation of the MF motion and AWP using systems of coupled differential equations in partial derivatives of Nernst–Planck–Poisson and Navier–Stokes (6–12) with boundary conditions (13–23), for the two-dimensional case, are presented in Figure 11.

Figure 11a shows the distribution of the concentration of charged particles in a cell with magnetic fluid in a plane perpendicular to the electrode, obtained by a computer simulation of a two-dimensional model. Figure 11a—the graph of the total concentration of C_1_ + C_2_ at a voltage of 8 V—shows a periodic structure of the concentration distribution, qualitatively consistent with the experimental studies, as shown in Figure 11b.

**Figure 12:**
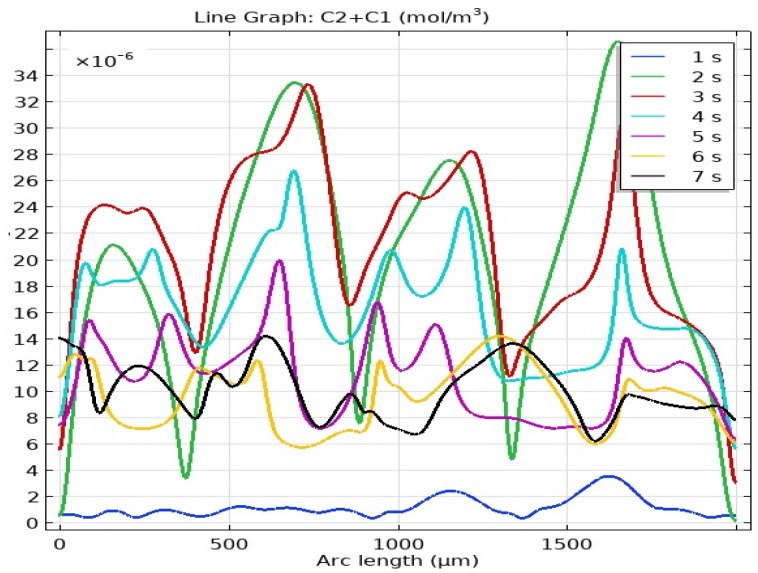
Graphs of the total concentration of C_1_ + C_2_ on the cathode surface at *t* = 1–7 s.

Graphs of the total concentration of C_1_ + C_2_ on the cathode surface at times *t* = 1–7 s. (Figure 12) fully correspond to concentric waves on the cell surface in a laboratory experiment, however, a qualitative agreement was achieved only for the three-dimensional model.

The result of studying the two-dimensional model shows that the appearance of periodic structures is associated with electroconvection, i.e., the motion of the magnetic fluid in the cell is caused by the action of an external electric field on the space charge regions induced by the same field.

Note that the third term, which is responsible for the convective transfer of particles, appears in Equation (6) only in the two-dimensional model. This term includes the electric ponderomotive volume force ($\vec{f}$) from the right-hand side of the Navier–Stokes Equations (10)–(12). This force acts on every point of the space charge.

The main result of the two-dimensional model, which distinguishes it from the one-dimensional one, is, firstly, the appearance of new periodic structures associated with the motion of the magnetic fluid inside the cell, and, secondly, the appearance of chaos at sufficiently high voltages (over 15 V) due to the fact that electroconvective vortices, colliding with each other, begin to behave chaotically.

The disadvantage of the two-dimensional model is the impossibility of identifying some types of waves (concentric and spiral waves), which can be done in three-dimensional modeling.

### 4.3. Three-Dimensional Model

**Figure 13:**
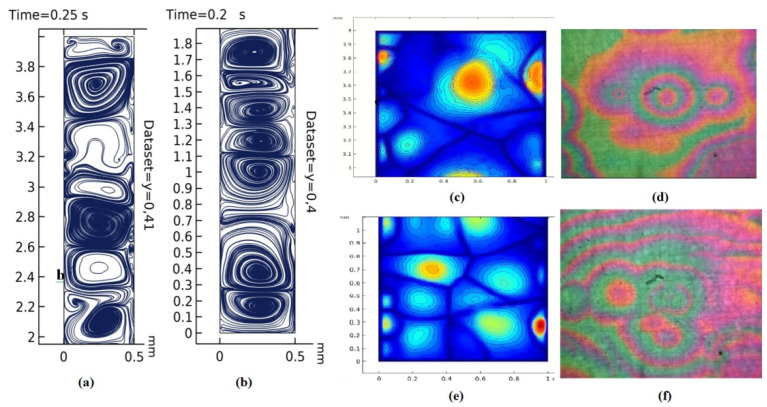
Comparison of the results of numerical simulation (**a**–**c**,**e**) and laboratory experiment (**d**,**f**): the appearance of concentric (**c**,**e**) and spiral-shaped waves (**a**,**b**) on the electrode surface. All scale bars are 1 cm.

**Figure 14:**
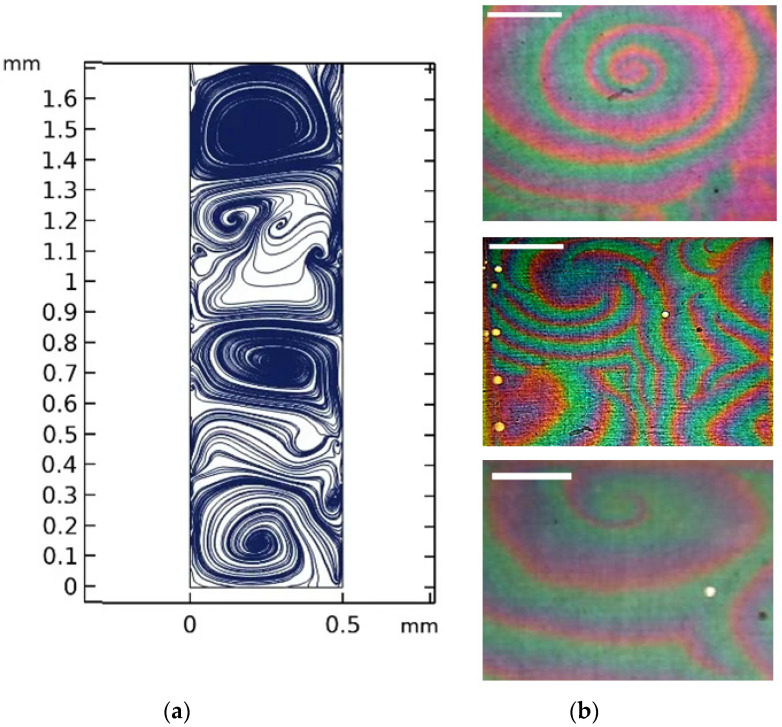
Comparison of numerical (**a**) and laboratory (**b**) experiments. Development of spiral waves (reverberators)—visualization of AWP in the plane of the electrode. All scale bars are 1 cm.

**Figure 15:**
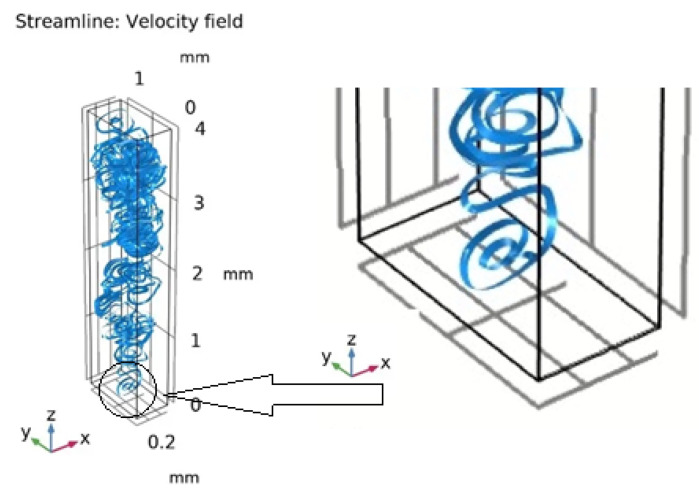
Development of a spiral wave inside the cell.

An implementation of two autowave modes, concentric waves (pacemakers) and spiral waves (Figure 13, Figure 14 and Figure 15), was obtained as a result of mathematical and computer three-dimensional modeling. Visual coincidence with the experimental results allows us to speak about the adequacy of the developed model.

The study of the three-dimensional model allowed us to develop the vortex motion of the liquid in the form of a spiral inside the cell (Figure 15).

It is especially necessary to consider the question of the timing of numerical experiments. The calculations of the one-dimensional model took a short time (several hours) and it is quite easy to study it. Calculations of a two-dimensional model are much more complicated and take from several days to weeks, but it is still possible to explore it. Calculations for a full-fledged three-dimensional model are extremely difficult at the limit of the capabilities of modern supercomputer systems and take months of calculations with a full processor load.

The three-dimensional model shows the real structures of the autowave process caused by the recharge of particles, such as spiral and concentric waves, which is impossible in a two-dimensional model. It is these structures that are observed experimentally. However, this model is not without its drawbacks, since not all structures found in the experiment can be simulated. This disadvantage is associated with the fact that the model is limited by the idea of charge exchange, and such processes as dissociation/recombination, the effect of impurity ions, and some other features of the process have not yet been taken into account.

Therefore, we can conclude that a three-dimensional model should be used to study the important and significant details of the process, a two-dimensional model should be used for less detailed studies, and a one-dimensional model is sufficient to identify general patterns such as oscillations, frequencies, and amplitudes.

## 5. Experimental Methods and Materials

In our experiments, we use a magnetic fluid [1] with magnetite (FeO·Fe_2_O_3_) as the dispersed phase and kerosene (aircraft TC1) as the carrier medium. The average particle size of magnetite is 10 nm. The stabilizer (surfactant) is oleic acid (CH_3_ (CH_2_)_7_CH=CH (CH_2_)_7_COOH). The concentration of magnetite in the samples of magnetic fluid used in the experiments is 3.2% vol (0.74·10^−3^ mol·m^−3^), relative dielectric constant ε = 2.1, and specific conductance σ = 3.8·10^−7^ Ohm^−1^·m^−1^ (measured at a frequency of 1000 Hz).

**Figure 16:**
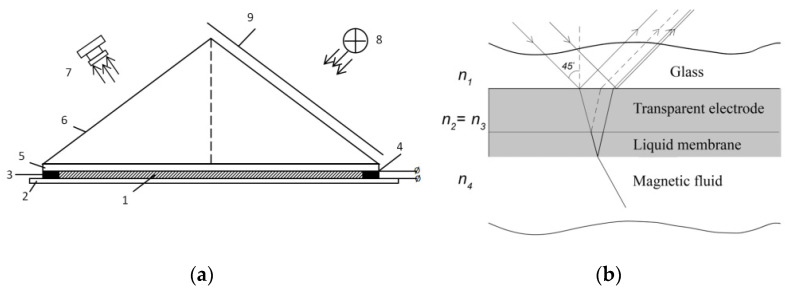
(**a**) Schematic of an experimental device for observing an autowave process: 1—magnetic fluid (MF); 2,5—glass with a transparent conductive coating (ITO); 3,4—polystyrene insulating gaskets; 6—rectangular isosceles prism; 7—camera; 8—light source, 9—frosted glass. (**b**) Schematic of the multilayer structure “glass-electrode–liquid membrane–magnetic fluid”. The path of the rays of light is shown by arrows.

The experiment was carried out on the setup shown in Figure 16a. The magnetic fluid is placed in a cell between two electrodes made of glass with a transparent conductive coating InSnO_2_ (indium tin oxide—ITO). The area of the electrodes is *S* = 30·40 mm^2^. The thickness of the glass is 4 mm, the thickness of the conductive coating is h_0_ = 160 ± 5 nm, the thickness of the magnetic fluid layer between the two electrodes is 50 μm.

The surface of the cell with the magnetic fluid is illuminated by a white LED. The rays reflected from the cell surface are recorded by a high-speed digital camera with a high resolution of 24.5 Mp Nikon Z6 II. Constant or pulsed voltage is applied to the electrodes. A rectangular prism is used for the spatial separation of the rays reflected from the boundaries of the ITO and the upper boundary of the glass, as well as for more effective visual observation of the processes occurring on the MF surface. Optical contact of the prism with glass is achieved by applying a thin immersion layer between them. Frosted glass was installed to scatter the incident light.

The lightwave falling on the ITO interferes with reflection (Figure 16b). The complex refractive indices and the thickness of the conductive coating (ITO) were measured using an SE 800 SENTECH spectroscopic ellipsometer. The refractive index of glass *n* = 1.52 and the refractive index of a conductive coating ${\hat{n}}_{2}=1.76\left(1+0.04i\right)$.

When voltage is applied to the electrodes, charged colloidal particles of magnetite move to the nearest electrode (the phenomenon of electrophoresis). A stable layer of densely packed particles of the dispersed phase—colloidal particles of magnetite—is formed at the surface of each of the electrodes. The properties of such a layer [16,24,26] differ significantly from the properties of a magnetic fluid in the cell far from the electrodes. The concentration of particles in the near-electrode layer is ~27–30% vol., which corresponds to a dense packing of particles. The refractive index of the magnetic fluid of this concentration is ${\hat{n}}_{4}=1.75\left(1+0.03i\right)$. The basic concentration of colloidal particles of magnetite is 3.2% vol, and the refractive index is ${\hat{n}}_{3}=1.45\left(1+0.01i\right)$.

The refractive indices of ITO and the near-electrode layer are almost similar. In an electric field, the thickness of the optically equivalent structure “ITO–near-electrode layer” changes. When determining the total thickness of such a two-layer structure, as well as the thickness of the layer, we follow the procedure described in [16]. In the two-layer structure “ITO conductive coating–near-electrode layer”, falling beams interference occurs. Using electric fields of different strengths, we can control the thickness of the near-electrode layer. At the same time, we observe a change in the color of the cell surface from green to crimson (the color of thin films). This electro-optical effect has been called electrically controlled interference [18].

**Figure 17:**
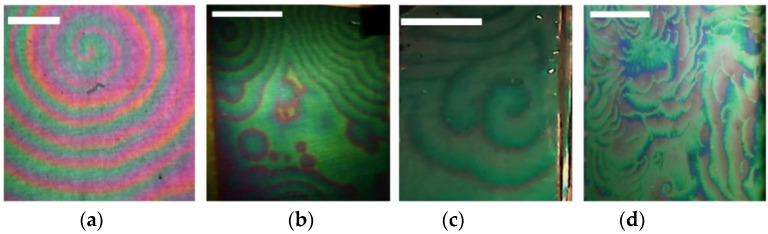
Autowaves in a cell with a magnetic fluid: (**a**) spiral, (**b**) concentric waves, (**c**) antispiral, (**d**) packet waves. All scale bars are 1 cm.

The visible color change of the layer is explained by the shift of the spectrum maximum due to the increase in the optical thickness of the “ITO-layer” structure. If the voltage on the electrodes is higher than the critical one (~12 V), the layer becomes unstable and the autowave process (AWP) begins [26,31,47]. In the course of the experimental studies, practically all types of known autowaves are observed: spirals, anti-spirals, concentric waves, packet waves, and autowaves with rectilinear propagation fronts (Figure 17). Video files with the movement of waves are available on https://disk.yandex.ru/d/GK7Y3uUaWujmuA (access date 29 January 2022).

## 6. Conclusions

A comprehensive experimental and theoretical study of the autowave process in a magnetic fluid is carried out in this paper. A thin layer, consisting of particles of the dispersed phase, is formed in a constant electric field at the boundary with the electrode. The thickness of the layer changes in an electric field, which is fixed by the method of electrically controlled interference. The mechanism of the autowave process is based on the interaction between the near-electrode layer and ions, injected from the electrode as a result of electrochemical reactions. The layer of close-packed particles serves as a membrane that prevents ions of the same charge sign with the electrode from entering the volume of the cell and recombining with ions of the opposite sign located in the near-electrode regions. A magnetofluid membrane has special properties: it forms in the field *U*_1_, collapses in the field *U*_2_, and its thickness depends on the magnitude of the external electric field. Due to the interaction of magnetic particles, the membrane does not mix with the other liquid in the cell.

A hierarchical system of mathematical models AWP is proposed. It consists of one-, two- and three-dimensional models based on general conservation laws, in which the idea of recharging MF particles in a liquid membrane is realized. Despite the fact that the models do not take into account chemical reactions on electrodes and dissociation–recombination reactions, it is possible to obtain results that qualitatively coincide with the experimental ones, as well as to investigate AWP parameters, such as frequency, amplitude, and others. An important result is that the characteristics of the wave process are not initially included in the models, however, the models demonstrate wave motion. This is the novelty of the proposed approach to AWP modeling.

The investigated models constitute the so-called hierarchy, since, on the one hand, they are self-sufficient for the modeling and analysis of AWP, and on the other hand, each previous one is an integral part of the next one and is a special case of the model of the next level. Therefore, first, the results of one-dimensional modeling are considered, the model is verified and analyzed, and then the two-dimensional and three-dimensional models are investigated. The results of each level are compared with each other in order to make sure they are adequate.

As a result of the implementation of the one-dimensional model, it is possible to obtain concentration waves, which show the correctness of the idea of particle recharge in a liquid membrane incorporated in the model. As a result of the implementation of the two-dimensional model, a periodic change in concentration was obtained, which showed a qualitative agreement with the experiment. As a result of three-dimensional modeling, vortices (spiral waves) and pacemakers (leading centers) were obtained, and it became possible to visualize the development of spiral waves inside the cell, which is impossible in a laboratory experiment.

Thus, we can conclude that the way to study such a complex phenomenon as the autowave process in a magnetic colloid was chosen correctly because, using only conservation laws, results were obtained that qualitatively correspond to laboratory experiments. The next step in the development of this study should be a quantitative agreement with the experiment by taking into account the influence of such phenomena as the electrical conductivity of a magnetic fluid, dissociation/recombination, and impurity and injected ions. This is the prospect of further research.

## Data Availability

Not applicable.

## Appendix A

### Appendix A.1. A Full Equation Set for 1D Model

System of equations:

$$\frac{\partial C_{i}}{\partial t}=-\frac{\partial j_{i}}{\partial x},\;i=1,2$$

$$j_{i}=-\frac{F}{RT_{0}}z_{i}D_{i}C_{i}\frac{d\varphi }{dx}-D_{i}\frac{dC_{i}}{dx},\;i=1,2$$

$$\frac{d^{2}\varphi }{dx^{2}}=-\frac{F}{\varepsilon_{r}}(z_{1}C_{1}+z_{2}C_{2})$$

Boundary and initial conditions:

$${\left(-\frac{F}{RT_{0}}D_{1}C_{2}\frac{d\varphi }{dx}-D_{1}\frac{dC_{2}}{dx}\right)|}_{x=0}=j_{1A},\;{\left(\frac{F}{RT_{0}}D_{2}C_{1}\frac{d\varphi }{dx}-D_{2}\frac{dC_{1}}{dx}\right)|}_{x=0}=j_{2A}$$

$$-{\left(-\frac{F}{RT_{0}}D_{1}C_{2}\frac{d\varphi }{dx}-D_{1}\frac{dC_{2}}{dx}\right)|}_{x=H}=j_{1K},-{\left(\frac{F}{RT_{0}}D_{2}C_{1}\frac{d\varphi }{dx}-D_{2}\frac{dC_{1}}{dx}\right)|}_{x=H}=j_{2K}$$

φ(t,0)=Δφ, φ(t,H)=0

$$C_{1}(0,x)=C_{10}(x),\;C_{2}(0,x)=C_{20}(x)$$

$$\varphi (0,x)=\varphi_{0}(x)=\frac{H-x}{H}\Delta \varphi$$

$$C_{1}(0,x)=C_{10}(0,x)=0.0074\cdot e^{-x/(0.01\cdot H)}\;mol\cdot m^{-3}$$

$$C_{2}(0,x)=C_{20}(0,x)=0.0074\cdot e^{-(H-x)/(0.01\cdot H)}\;mol\cdot m^{-3}$$

### Appendix A.2. A Full Equation Set for 2D Model

System of equations:

$${\vec{j}}_{i}=-\frac{F}{RT}z_{i}D_{i}C_{i}\vec{E}-D_{i}\nabla C_{i}+C_{i}\vec{V},\;i=1,2$$

$$\frac{\partial C_{i}}{\partial t}=-div{\vec{j}}_{i},\;i=1,2$$

$$\varepsilon_{r}\Delta \varphi =-F\left(z_{1}C_{1}+z_{2}C_{2}\right),$$

$$\vec{I}=F\left(z_{1}{\vec{j}}_{1}+z_{2}{\vec{j}}_{2}\right)$$

$$\frac{\partial \vec{V}}{\partial t}+\left(\vec{V}\nabla \right)\vec{V}=-\frac{1}{\rho_{0}}\nabla P+\nu \Delta \vec{V}+\frac{1}{\rho_{0}}\vec{f},$$

$$div(\vec{V})=0,$$

$$\vec{f}=\rho \vec{E}$$

$$\frac{\partial C_{i}}{\partial t}=\frac{F}{RT}z_{i}D_{i}div\left(C_{i}\nabla \varphi \right)-D_{i}\Delta C_{i}-div\left(C_{i}\vec{V}\right),\;i=1,2.$$

Boundary and initial conditions:

$$V_{x}\left(0,x,y\right)=V_{y}\left(0,x,y\right)=0$$

Conditions at the anode (*x* = 0):

$${\left(-\frac{F}{RT_{0}}D_{1}z_{1}C_{2}\frac{d\varphi }{dx}-D_{1}\frac{dC_{2}}{dx}\right)|}_{x=0}=j_{1A},\;{\left(-\frac{F}{RT_{0}}D_{2}z_{2}C_{1}\frac{d\varphi }{dx}-D_{2}\frac{dC_{1}}{dx}\right)|}_{x=0}=j_{2A}$$

φ(t,0,y)=α

$$-\vec{n}\cdot \vec{V}(t,0,y)=0\;$$

Conditions at the cathode (*x* = *H*):

$${\left(-\frac{F}{RT_{0}}D_{1}z_{1}C_{2}\frac{d\varphi }{dx}-D_{1}\frac{dC_{2}}{dx}\right)|}_{x=H}=j_{1k},\;{\left(-\frac{F}{RT_{0}}D_{2}z_{2}C_{1}\frac{d\varphi }{dx}-D_{2}\frac{dC_{1}}{dx}\right)|}_{x=H}=j_{2K}$$

φ(t,H,y)=0

$$-\vec{n}\cdot \vec{V}(t,H,y)=0\;$$

Condition on insulators (*y* = 0, *y* = L):

$$-\vec{n}\cdot {\vec{j}}_{i}=0,\;i=1,2\;$$

$$-\vec{n}\cdot \nabla \varphi =0.$$

Initial conditions:

$$C_{i}(0,x,y,z)=C_{i0}\;i=1,2$$

$$\varphi (0,x,y)=\alpha t-\frac{\alpha tx}{H}$$

$$C_{1}(0,x,y)=C_{10}(0,x,y)=0.0074\cdot e^{-x/(0.01\cdot H)}\;mol\cdot m^{-3}$$

$$C_{2}(0,x,y)=C_{20}(0,x,y)=0.0074\cdot e^{-(H-x)/(0.01\cdot H)}\;mol\cdot m^{-3}$$

$$C_{1}(0,x,y)=C_{10}(0,x,y)$$

$$C_{2}(0,x,y)=C_{20}(0,x,y)$$

### Appendix A.3. A Full Equation Set for 3D Model

System of equations:

$${\vec{j}}_{i}=-\frac{F}{RT}z_{i}D_{i}C_{i}\vec{E}-D_{i}\nabla C_{i}+C_{i}\vec{V},\;i=1,2,$$

$$\frac{\partial C_{i}}{\partial t}=-div{\vec{j}}_{i},\;i=1,2,$$

$$\varepsilon_{r}\Delta \varphi =-F\left(z_{1}C_{1}+z_{2}C_{2}\right),$$

$$\vec{I}=F\left(z_{1}{\vec{j}}_{1}+z_{2}{\vec{j}}_{2}\right)$$

$$\frac{\partial \vec{V}}{\partial t}+\left(\vec{V}\nabla \right)\vec{V}=-\frac{1}{\rho_{0}}\nabla P+\nu \Delta \vec{V}+\frac{1}{\rho_{0}}\vec{f},$$

$$div(\vec{V})=0,$$

$$\vec{f}=\rho \vec{E}$$

Boundary and initial conditions:

$$V_{x}\left(0,x,y,z\right)=V_{y}\left(0,x,y,z\right)=0.$$

$${\left(-\frac{F}{RT_{0}}D_{1}z_{1}C_{2}\frac{\partial \varphi }{\partial x}-D_{1}\frac{\partial C_{2}}{dx}\right)|}_{x=0}=j_{1A}$$

$${\left(-\frac{F}{RT_{0}}D_{2}z_{2}C_{1}\frac{\partial \varphi }{\partial x}-D_{2}\frac{\partial C_{1}}{dx}\right)|}_{x=0}=j_{2A}$$

$${\left(-\frac{F}{RT_{0}}D_{1}z_{1}C_{2}\frac{\partial \varphi }{\partial x}-D_{1}\frac{\partial C_{2}}{dx}\right)|}_{x=H}=j_{1K}$$

$${\left(-\frac{F}{RT_{0}}D_{2}z_{2}C_{1}\frac{\partial \varphi }{\partial x}-D_{2}\frac{\partial C_{1}}{dx}\right)|}_{x=H}=j_{2\;K}$$

φ(t,0,y,z)=α

φ(t,H,y,z)=0

$$-\vec{n}\cdot {\vec{j}}_{i}=0,\;i=1,2\;$$

$$C_{1}(0,x,y,z)=C_{10}(0,x,y,z)=0.0074\cdot e^{-x/(0.01\cdot H)}\;mol\cdot m^{-3}$$

$$C_{2}(0,x,y,z)=C_{20}(0,x,y,z)=0.0074\cdot e^{-(H-x)/(0.01\cdot H)}\;mol\cdot m^{-3}$$

$$\varphi (0,x,y,z)=\alpha -\frac{\alpha x}{H}$$
